# Relationship between paravertebral muscle degeneration and spinal-pelvic sagittal parameters in patients with lumbar disc herniation

**DOI:** 10.1038/s41598-023-50836-4

**Published:** 2024-01-02

**Authors:** Tianlian Bao, Chunmei Wang, Yongjiang Wang, Tiantian Wang, Qingxin Zhang, Feng Gao, Hao Liu, Xiaoyang Tao, Gang Gao, Tinxin Zhang, Wupeng Yang, Keyu Zhao

**Affiliations:** 1 Orthopaedics of Traditional Chinese Medicine, The Traditional Chinese Medicine Hospital of Dongyang, Dongyang, 322100 Zhejiang People’s Republic of China; 2 The Ordos Clinic Medical College, Inner Mongolia Medical University, 23 Ekin Hollow West Street, Ordos City, 017000 Inner Mongolia Autonomous Region People’s Republic of China; 3 Department of Orthopedics, Ordos Central Hospital, Ordos, 017000 Inner Mongolia Autonomous Region People’s Republic of China

**Keywords:** Health care, Medical research

## Abstract

Lumbar disc herniation (LDH) is a clinically common degenerative disease of the spine, and spinal–pelvic sagittal balance and paravertebral muscle degeneration have been a research focus in recent years. To explore the relationship between the degeneration of paravertebral muscle and the changes in the spinal–pelvic sagittal parameters in LDH patients, 105 LDH patients (experimental group) and 63 healthy volunteers (control group) hospitalized in Ordos Central Hospital from January 2020 and January 2023 were included as study subjects. All the patients underwent lumbar magnetic resonance imaging and spinal X-ray using uniform criteria. The correlation between the paravertebral muscle and sagittal–pelvic sagittal parameters of the patients with LDH was obtained from two imaging examinations, and the data were organized and grouped to explore the correlation between these parameters. No significant difference in general data existed between the groups (P > 0.05). In the L4/5 LDH patients group, the ratio of fat infiltration (FIR) in the healthy side [multifidus (MF) and erector spinae (ES)] was negatively correlated with the lumbar lordosis (LL) (r = −0.461, r = −0.486, P < 0.05). The relative cross-sectional area (RCSA) of the bilateral MF was positively correlated with the pelvic tilt (r = 0.549, r = 0.515, P < 0.05). The bilateral ES RCSA was negatively correlated with the sagittal vertical axis (r = −0.579, r = −0.621, P < 0.05). A positive correlation existed between the RCSA and thoracic kyphosis in the healthy side ES (r = 0.614, P < 0.05). In the L5/S1 LDH patients group, a negative correlation existed between the FIR and LL in the healthy side ES (r = −0.579, P < 0.05). Thus, the paravertebral muscle parameters were correlated with the spinal–pelvic sagittal parameters in the patients with LDH.

## Introduction

People’s exploration of diseases is deepening with the rapid advance of the times and the development of science and technology. As one of the most common diseases among Chinese people, the lifetime prevalence rate of low back pain is as high as 84%. Clinically, lumbar disc herniation (LDH) is one of the common causes of low back pain^1^. Furthermore, young people are slowly becoming the main population of this disease. Numerous studies have been conducted on LDH at home and abroad. Park et al.^2^ found that the degree of paravertebral muscle fat infiltration in LDH patients is higher than that in normal people, exhibiting different degrees of atrophy and reduced muscle function. Yaltɩrɩk et al.^3^ confirmed from a neurological perspective that the degeneration of the paravertebral muscle in LDH patients is aggravated by nerve injury (direct compression or inflammatory stimulation). The spine is mainly composed of the vertebral body, the intervertebral disc, vertebral facet joints and ligaments, and the paravertebral muscle, which is responsible for regulating and maintaining the balance of the spine. Many parameters of the spine’s balance have been quantitatively processed. Previous studies have shown that lumbar disc herniation may be associated with lumbar segmental instability^4^. Abelin-Genevois et al.^5^ elaborated the parameters of the spine and the pelvis in detail. Barrey et al.^6^ found that patients with degenerative LDH have smaller lumbar lordosis (LL) and thoracic kyphosis (TK) values than the normal population, indicating that the spinal curvature of the patients gradually disappears and becomes straight, showing an obvious imbalance. Ragnics and Endo et al.^7,8^ found that the pelvic tilt (PT) and the sagittal vertical axis (SVA) were larger and the LL and the sacral slope (SS) were smaller in patients with degenerative LDH than those in healthy people, while the pelvic incidence (PI) values have no significant difference. In recent years, scholars have paid great attention to the changes of the paravertebral muscle in degenerative diseases of the lumbar spine. Hiyama et al.^9–11^ respectively studied the relationship between paravertebral muscle degeneration and spine–pelvis parameters from the perspective of degenerative diseases, such as degenerative kyphosis, and normal people of different ages. Few studies on patients with LDH have been conducted. Although surgery for lumbar disc herniation has a good effect on symptomatic lumbar instability^12^, many patients still have recurrent or long-term chronic low back pain complications after surgical treatment, which may be related to the failure of open surgery to maintain spinal stability due to paraspinal muscle injury. In this study, we combined the research methods of the above scholars and our own research conditions to analyze the correlation between the lumbar paravertebral muscle mass and spinal–pelvic sagittal position parameters of LDH patients by measuring the paravertebral muscle and spinal–pelvic sagittal position parameters of LDH patients to provide a scientific basis for basic disease assessment and the personalized diagnosis, treatment, and rehabilitation of LDH patients.

## Materials and methods

### Sample size calculation

T-test and correlation analysis were adopted in this study design. Therefore, efficacy tests were conducted on T-test and correlation analysis respectively. The results showed that, in the T-test, the allowable error was assumed to be 0.8, the significance was set to be 0.05, and the efficacy was set to be 90%. A total of 102 samples were required for the three groups. In the correlation analysis, the significance was set at 0.05, the efficacy at 90%, and the two-sided test was adopted. Finally, the minimum sample size meeting the above conditions was 47 cases (Table 1).

**Table 1:** Power analysis of T-test and correlation.

Method	d	Sig.level	Power	Alternative	N	Total N
t-test	0.8	0.05	0.9	two.sided	33.83	102
Correlation	–	0.05	0.9	two.sided	46.91	47

### Study design

A total of 168 patients and healthy volunteers with LDH who were admitted to Ordos Central Hospital for treatment between January 2020 and January 2023 were included. Among all the patients (n = 168) who participated in the present study, there were 90 men and 78 women, ranging in age from 36 to 57 years old, including 105 patients in the experimental group (patients with LDH) and 63 patients in the control group (healthy volunteers). The present study was approved by the Ethics Committee of Ordos Central Hospital (Ordos, China). Informed consent was obtained from all subjects and/or their legal guardians for the present study. Inclusion criteria: (1) patients with unilateral single-segment LDH diagnosed by two deputy directors or above; (2) patients with segment L4/5 or L5/S1; (3) patients with a disease course of 3 months or more; (4) patients with low back pain combined with lower extremity radial pain; (4) complete clinical data (lumbar MRI, spinal X-ray); (5) patients with relevant informed consent; Exclusion criteria: (1) Patients with a personal history of long-term hormone use, various types of neurological or muscular diseases resulting in lumbar paravertebral muscle atrophy; (2) patients with a history of surgical treatment of lumbar diseases, or a history of trauma and paravertebral myopathy; (3) patients with multi-segmental and high lumbar disc herniation, (4) patients with coronary disequilibrium, (5) patients with radiating pain of lower limb or low back caused by non-LDH diagnosed by two or more physicians; (6) serious other medical and surgical diseases cannot cooperate with the examination; (7) patients with mental illness, pregnant women, communication difficulties or cognitive impairment. The inclusion criteria for the control group were individuals with lumbar MRI and spinal X-ray. No history of any lumbar disease, low back pain, radiation pain in the lower extremities, and neurogenic claudication. The exclusion criteria were the same as in the experimental group. All enrolled patients and healthy volunteers were recorded by name, age, gender, weight, height, etc., and MRI and full-length anterior and lateral spinal X-ray were performed according to actual conditions and unified standards.

### Imaging examination

X-rays of the spine were performed using the universal digital radiography system (General Medical Merate S.p.A.). When X-ray is taken, the radial bulb moves vertically in line with the detector, and the corresponding image is obtained through several exposures. The obtained image is automatically splicing by the device software to obtain the lateral image of the spine. The relevant parameters were measured mainly by spinal lateral X-ray.

Magnetic resonance imaging (MRI) was performed with Signa HDxt 3.0T magnetic resonance scanner (GE Healthcare) and integrated cervical, thorax and lumbar coils. When taking lumbar MRI, the scanning and positioning lines were placed at the umbilical level. All groups were supine with advanced head. Sagittal fat inhibition FSE T2WI: TE = 42.72, TR = 3246, DFOV = 31 × 31 cm, layer thickness = 4 mm, layer spacing = 5 mm, excitation frequency (NEX) = 2, cross section T2WI: TE = 123.66, TR = 2854, DFOV = 20 × 20 cm, layer thickness = 4 mm, layer spacing = 5 mm, excitation times (NEX) = 2. In this paper, the position and degree of nucleus pulposus protrusion were observed from various angles in axial and sagittal positions, the responsible lesions were identified in combination with the patient's medical history and signs, and the cross-sectional MRI T2-weighted images corresponding to the lesions were selected and transmitted to ImageJ software for measurement.

### Evaluation indicators

**Figure 1:**
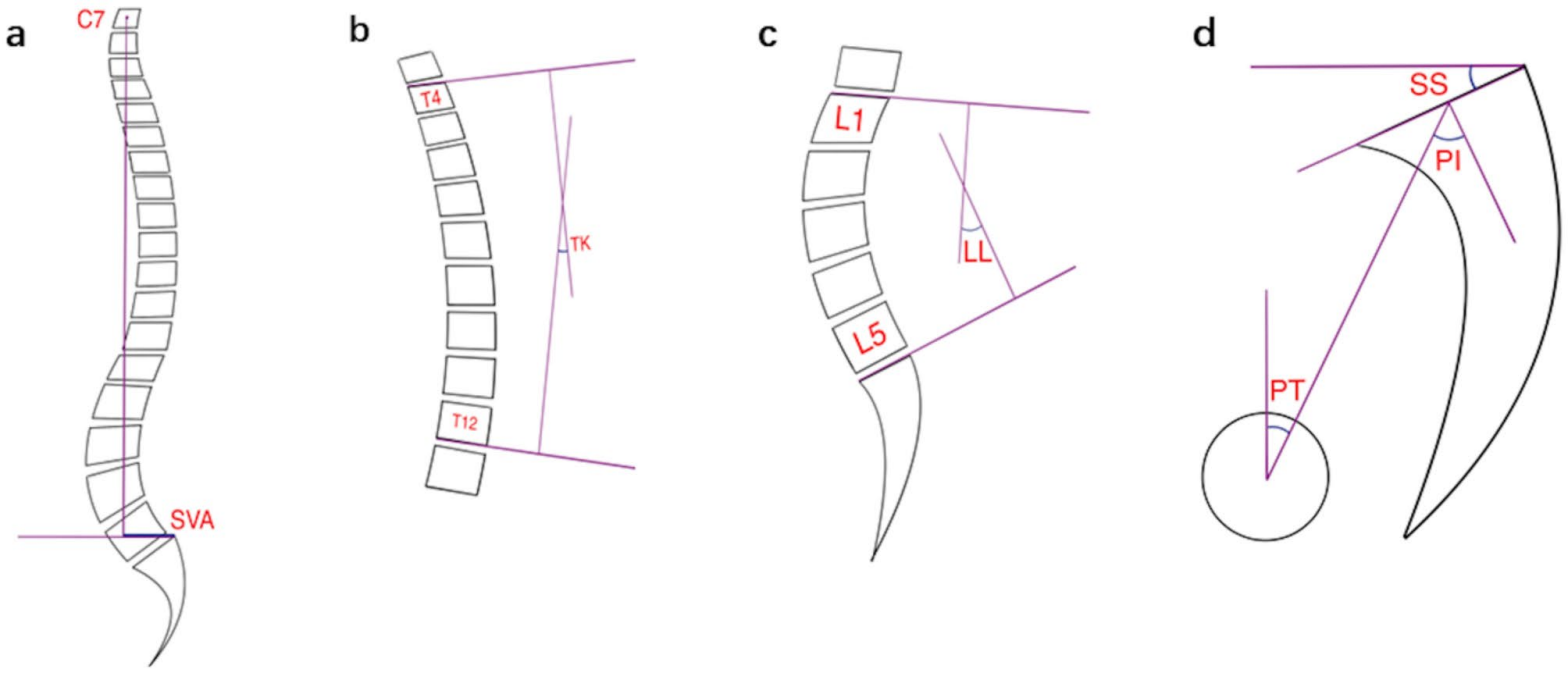
Measurement diagram developed using Surgimap. (**a**) SVA, the distance between the plumb line of the seventh cervical vertebra and the posterior upper angle of the first sacrum. LL, lumbar lordosis; (**b**) TK, the angle formed by the tangent of the upper edge of the T4 vertebral body and the tangent of the lower edge of the T12 vertebral body. (**c**) LL, the angle formed by the tangent of the upper edge of the L1 vertebral body and the tangent of the upper endplate of the S1 vertebral body. (**d**) PT, passing through the middle of the upper endplate of S1, a straight line between the point and the midpoint of the line connecting the centers of the bilateral femoral heads was added in order to exhibit the angle formed by the straight line and the long axis of the body. SS, the angle formed by the tangent line of the upper endplate of S1 and the horizontal line. PI, a straight line through the midpoint of the line connecting the midpoint of the upper endplate of S1 and the center of the bilateral femoral heads was added. The angle formed by the vertical line of the upper endplate of S1 is depicted. *SVA* sagittal vertical axis, *TK* thoracic kyphosis, *LL* lumbar lordosis, *PT* pelvic tilt, *SS* sacral slope, *PI* pelvic incidence.

**Figure 2:**
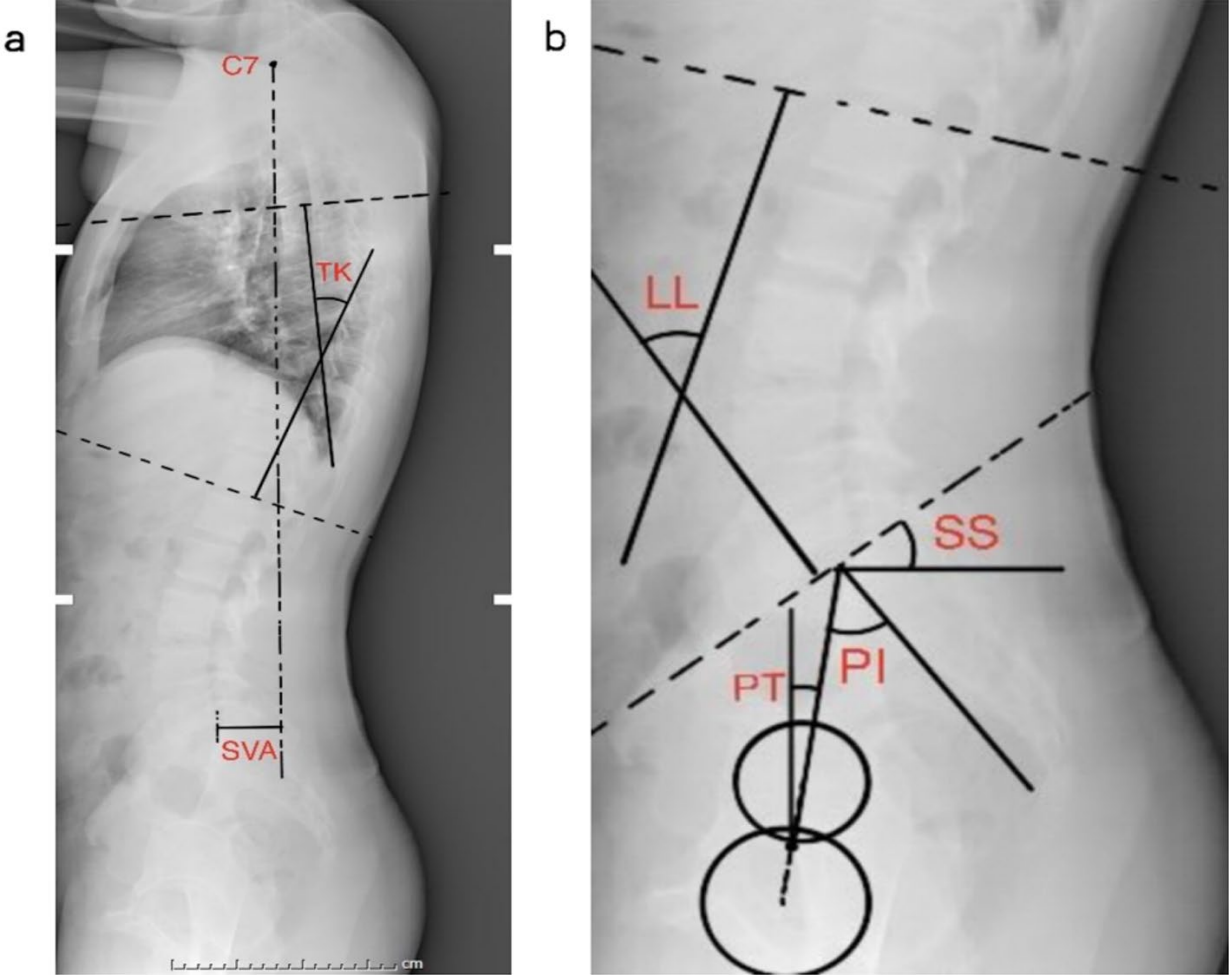
Spine-pelvic parameters measured using Surgimap. (**a**) TK, the angle formed by the tangent of the upper edge of the T4 vertebral body and the tangent of the lower edge of the T12 vertebral body. SVA, the distance between the plumb line of the seventh cervical vertebra and the posterior upper angle of the first sacrum. (**b**) LL, the angle formed by the tangent of the upper edge of the L1 vertebral body and the tangent of the upper endplate of the S1 vertebral body. PT, passing through the middle of the upper endplate of S1, a straight line between the point and the midpoint of the line connecting the centers of the bilateral femoral heads was created, and the angle formed by the straight line and the long axis of the body is depicted. SS, the angle formed by the tangent line of the upper endplate of S1 and the horizontal line. PI, a straight line through the midpoint of the line connecting the midpoint of the upper endplate of S1 and the center of the bilateral femoral heads was added. The angle formed by the vertical line of the upper endplate of S1 is revealed. *LL* lumbar lordosis, *TK* thoracic kyphosis, *PT* pelvic tilt, *SS* sacral slope, *PI* pelvic incidence, *SVA* sagittal vertical axis.

Spine–pelvic Parameters: The Surgimap software is a free computer program (v2.3.2.1, USA) that combines spine-related measurement and surgical planning tools to provide a practical graphical approach to balancing related parameters, such as spine, pelvis, and lower limbs. Lafage et al.^11^ further confirmed that the Surgimap software has advantages, such as short processing time, less error, and convenient data storage, over traditional manual methods in measuring relevant parameters and is thus suitable for clinical use. Standard full-length anterior and lateral spine positions were obtained from the imaging department of our hospital in JPG format and imported into the Surgimap software. The spinal and pelvic sagittal position parameters of the experimental group and control group were measured by the two or more attending spinal surgeons according to the corresponding operating standards (Figs. 1 and 2).

**Figure 3:**
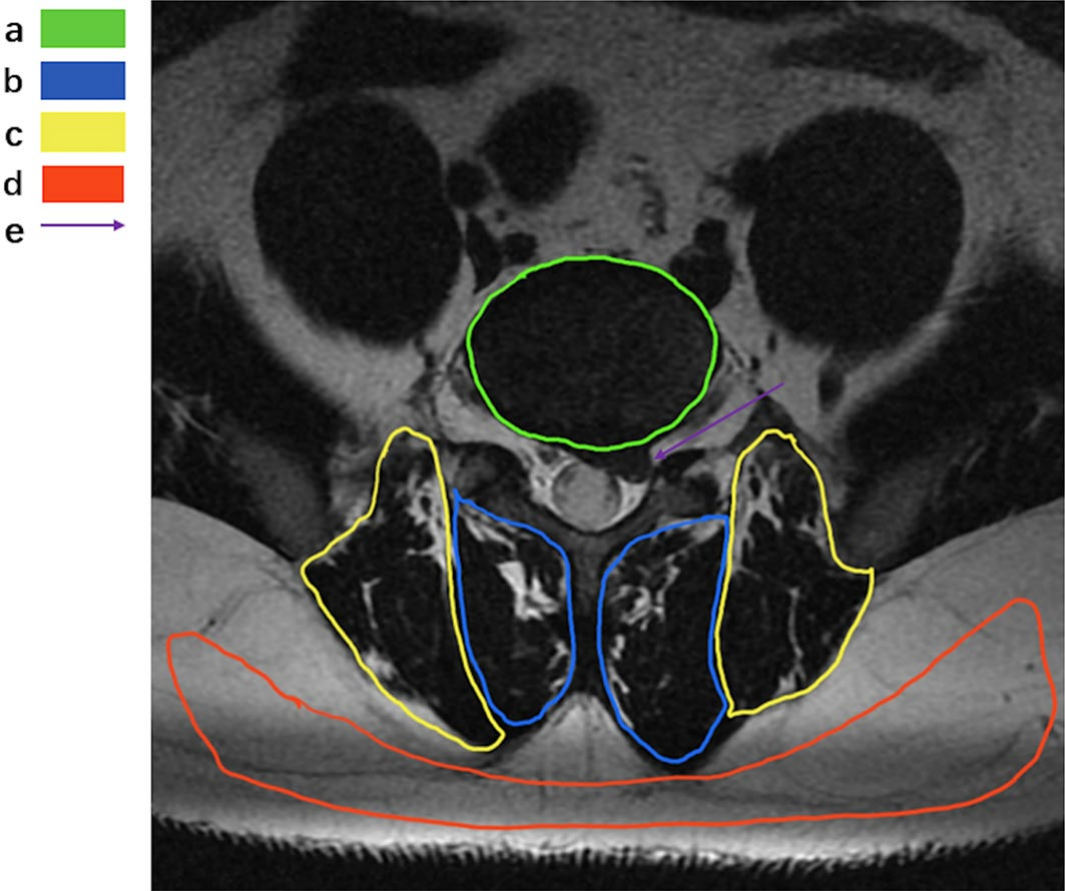
Example of the area of interest defined using ImageJ. (**a**) CSA of the target vertebral body is indicated by green; (**b**) multifidus-CSA by blue; (**c**) erector spinae CSA is indicated by yellow; (**d**) subcutaneous fat range by red; (**e**) the position of the disc protrusion is indicated by the purple arrow. *CSA* cross-sectional area.

**Figure 4:**
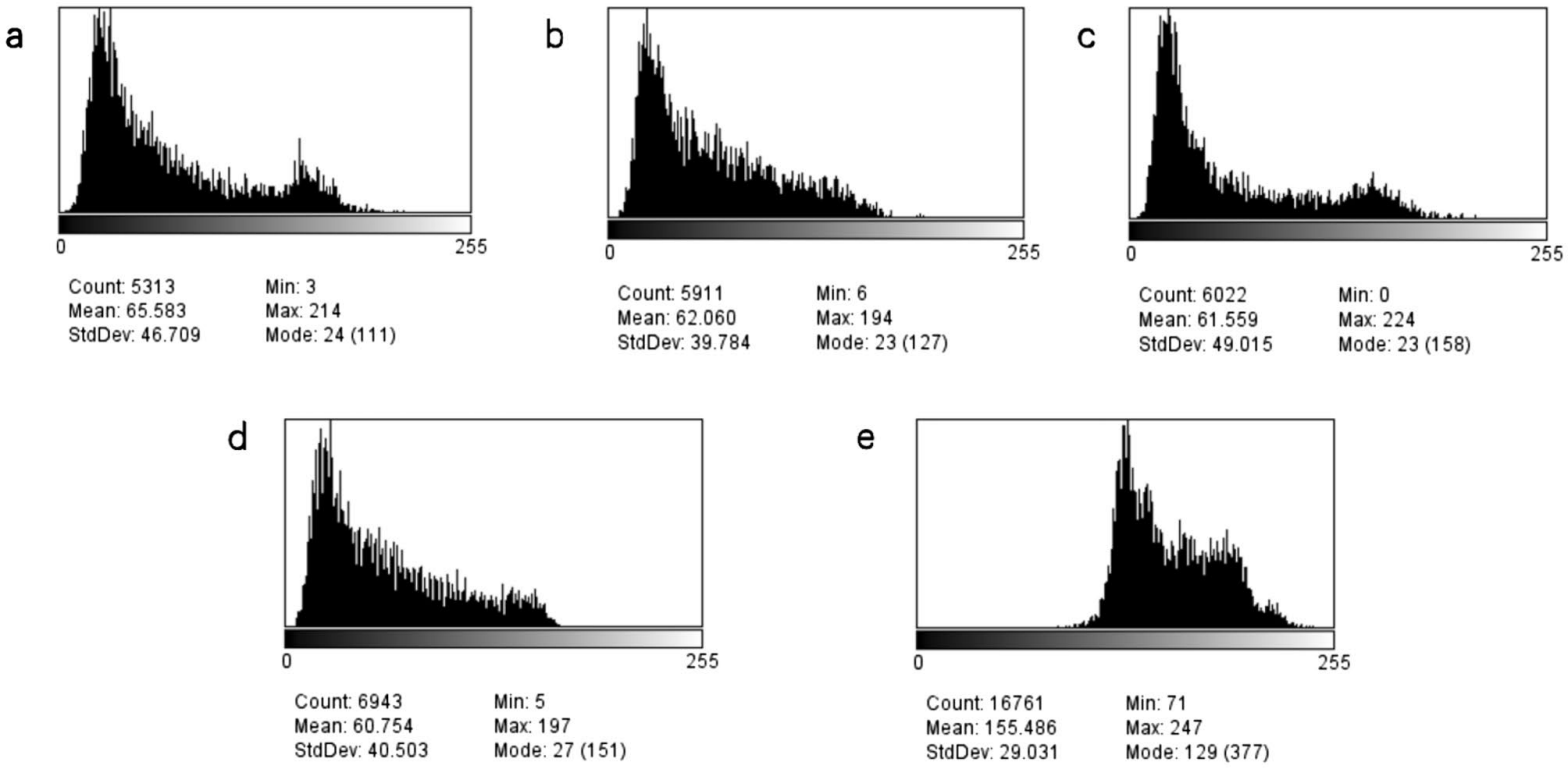
Grey-scale map of paravertebral muscle and subcutaneous fat constructed using ImageJ. (**a**) Right MF, (**b**) right ES, (**c**) left MF, (**d**) left ES, (**e**) subcutaneous fat. *MF* multifidus, *ES* erector spinae.

**Figure 5:**
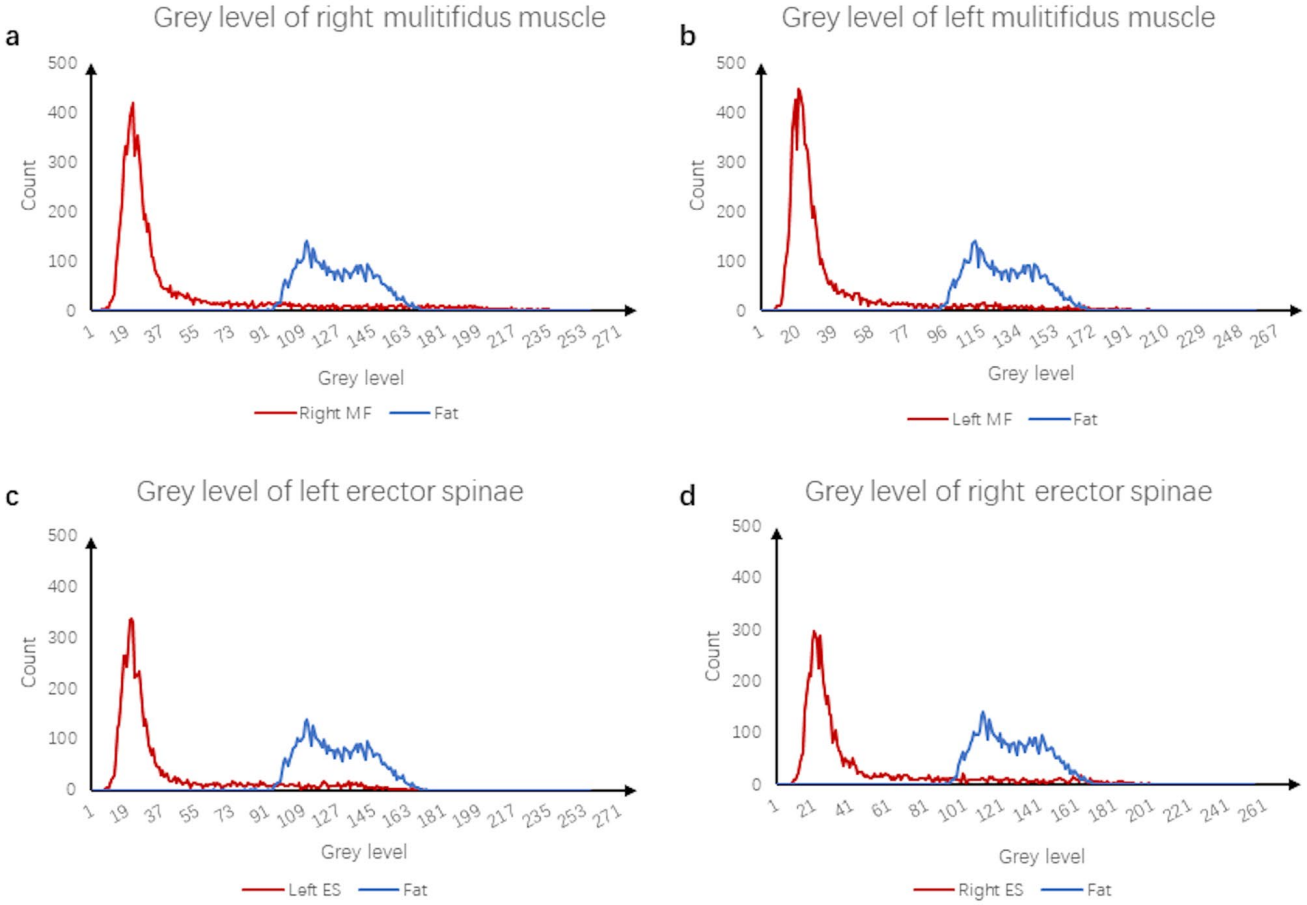
Gray value distribution line plot. (**a**) Blue line, Right MF muscle gray distribution; red, subcutaneous fat gray distribution. (**b**) Blue line, Left MF muscle gray distribution; red, subcutaneous fat gray distribution. (**c**) Blue line, Right ES muscle gray distribution; red, subcutaneous fat gray distribution. (**d**) Blue line, left ES muscle gray distribution; red, subcutaneous fat gray distribution. *MF* multifidus, *ES* erector spinae.

Paravertebral muscle parameters: ImageJ (v1.53c; National Institutes of Health) is a kind of public image processing and analysis software developed and designed by a number of American scientists using a proprietary language based on Java language. It supports DICOM format image processing, adapts to the data collected by the imaging department of our hospital, and has functions such as editing, processing, analysis, saving and even printing. The two attending spinal surgeons are proficient in the measurement of various indicators and collect corresponding data from the images. It should be noted that the two physicians only perform data measurement and do not participate in the design of experiments, disease diagnosis and data analysis. In Image J software, the region of interest is drawn on the selected image (Fig. 3) to obtain the corresponding cross-sectional area of bilateral paraveraveral muscles and the cross-sectional area of the upper vertebral body of the responsibility segment, and then the relative value is calculated: relative cross-sectional area (RCSA) = paraveraveral muscle area/area of the upper vertebral body of the responsibility segment × 100%. The software's threshold technology was further used to measure the gray values of paravertebral muscle and subcutaneous fat (Fig. 4), and the gray values of paravertebral muscle and subcutaneous fat were imported into Excel to further draw the line chart of gray value distribution, and then analyzed (Fig. 5). The ratio of the total gray value of the superimposed portion of the two-fold line plot to the total gray value of the paravertebral muscle indicates the fatty infiltration ratio (FIR) of the paravertebral muscle.

### Statistical analysis

Statistical analysis was performed using IBM SPSS Statistics (v.25.0; IBM Corp.). The counting data is represented as a ratio and described by the mean ± standard deviation. All the values were reserved after two decimal places, and the test level was 0.05. Pearson’s chi-squared test was used to compare the differences of the groups in gender distribution (two-tailed tests). One-way ANOVA was used to compare the differences in age and body mass index (BMI) among the groups (one-tailed tests). Independent or paired samples Student’s t-test was used to compare the differences between the experimental and control groups in MF and ES (two-tailed tests). Independent samples Student’s t-test was used to compare the differences between the experimental and control groups in spinal and pelvic sagittal position parameters (two-tailed tests). In the statistical analysis of correlation, the normality test result shows that P < 0.05, and the data did not follow normal distribution, so the Spearman rank test was used (two-tailed tests).

### Reliability analysis

Kendall coordination coefficient and ICC group correlation coefficient were used to evaluate the reliability of the two repeated measurement data respectively. The results showed that the minimum values of Kendall coordination coefficient and ICC group correlation coefficient in each measurement index were 0.846 (Z = 9.202, P < 0.001) and 0.967 (95% CI 0.873 ~ 0.987, P < 0.001), and the maximum values were 0.976 (Z = 10.601, P < 0.001) and 0.999 (95% CI 0.998 ~ 0.999), indicating a good consistency between the two measurements (Table 2).

**Table 2:** Reliability analysis of repeated measurements.

Variables	Kendall coordination coefficient			ICC group correlation coefficient			
	Kendall	Z.value	P.value	ICC	Lower.CI	Upper.CI	P value
Right MF FSF	0.974	10.601	0.000	0.998	0.966	0.999	0.000
Left MF FSF	0.964	10.488	0.000	0.997	0.966	0.999	0.000
Right ES FSF	0.916	9.966	0.000	0.993	0.975	0.997	0.000
Left ES FSF	0.853	9.280	0.000	0.989	0.982	0.994	0.000
Right MF CSA	0.919	10.008	0.000	0.995	0.992	0.997	0.000
Left MF CSA	0.918	9.993	0.000	0.992	0.987	0.996	0.000
Right ES CSA	0.935	10.177	0.000	0.995	0.992	0.997	0.000
Left ES CSA	0.935	10.177	0.000	0.997	0.996	0.998	0.000
Upper vertebral body CSA	0.947	10.304	0.000	0.999	0.998	0.999	0.000
SVA	0.978	10.644	0.000	0.999	0.994	1.000	0.000
TK	0.941	10.241	0.000	0.997	0.994	0.998	0.000
LL	0.857	9.329	0.000	0.985	0.968	0.992	0.000
PI	0.881	9.591	0.000	0.991	0.985	0.995	0.000
PT	0.926	10.078	0.000	0.989	0.882	0.997	0.000
SS	0.846	9.202	0.000	0.967	0.873	0.987	0.000

### Ethics approval and consent to participate

All methods were carried out in accordance with the relevant guidelines and regulations. All experimental protocols were approved by the Ethics Committee of Ordos Central Hospital, Inner Mongolia Medical University (Ordos, China). Informed consent was obtained from all the subjects and/or their legal guardians for the present study.

## Results

### Analysis of general data results

A total of 168 subjects were included in this study, namely, 105 in the experimental group and 63 in the control group. The mean age of subjects in the experimental group is 45.74 ± 11.02 years in the L4/5 group and 44.75 ± 8.91 years in the L5/S1 group. The ages of the subjects ranged from 36 to 57 years, with the mean age of 44.09 ± 9.66 years. No significant differences in age, sex, and BMI was observed among the three groups (P > 0.05) (Table 3).

**Table 3:** General data analysis.

General date	Control group	Experimental group		*χ*^2^/F	P
–	–	LDH (L4/5)	LDH (L5/S1)	–	–
Male, n (%)	27 (42.86)	36 (63.16)	27 (56.25)	5.152	0.076
Female, n (%)	36 (57.14)	21 (36.84)	21(43.75)	–	–
Age, years	42.1 ± 8.97	45.74 ± 11.02	44.75 ± 8.91	0.755	0.475
Body mass index (kg/m^2^)	22.95 ± 2.32	25.1 ± 4.32	23.84 ± 3.22	2.031	0.141

### Analysis of paravertebral muscle parameters

The results of the comparison between the control and experimental groups show no significant differences in the FIR and RCSA of the two sides of the control group (P > 0.05).

L4/5 patient group: The MF (or ES) FIR on the diseased side of the LDH patients group was statistically significant compared with that on the healthy side of the LDH patients group, P < 0.05. The MF (or ES) FIR on the diseased side of the LDH patients group was statistically significant compared with that of the ipilateral muscle of the subjects in the control group (P < 0.05). When the right side of the experimental group was the healthy side (or diseased side), the MF RCSA was lower than that of the ipilateral ES, P < 0.05. When the left side of the experimental group was the healthy side, the left MF RCSA was lower than that of the ipilateral ES, P < 0.05. When the left side was the diseased side, no significant difference in RCSA existed between the left MF and the ipsilateral ES (P > 0.05). The right MF RCSA in the control group was lower than that of the ipilateral ES, P < 0.05.

L5/S1 patient group: The MF (or ES) FIR on the diseased side of the LDH patients group was statistically significant compared with that on the healthy side of the LDH patients group, P < 0.05. The MF (or ES) FIR on the diseased side of the LDH patients group was statistically significant compared with that of the ipilateral muscle of the subjects in the control group (P < 0.05). When the right side of the experimental group was the healthy side (or diseased side), the MF RCSA was higher than that of the ipilateral ES, P < 0.05. When the left side of the experimental group was the healthy side, the left MF RCSA was higher than that of the ipilateral ES, P < 0.05. When the left side was the diseased side, no significant difference in RCSA existed between the left MF and the ipsilateral ES (P > 0.05). In the control group, the MF RCSA was higher than that of the ipsilateral ES (P < 0.05) (Tables 4 and 5).

**Table 4:** Paravertebral muscle parameters in LDH patients (L4/5) and control group.

	LDH patient group (L4/5)		Control group	
	Diseased side	Healthy side	Right	Left
MF-FIR, %	–	–	–	–
Right	26.39 ± 3.7★▲	19.23 ± 7.09	15.95 ± 3.82	17.89 ± 3.86
Left	22.5 ± 4.11★▲	16.43 ± 3.95	15.95 ± 3.82	17.89 ± 3.86
ES FIR (%)	–	–	–	–
Right	24.49 ± 10.47★▲	14.76 ± 4.23	15.70 ± 2.26	16.53 ± 1.00
Left	25.93 ± 8.74★▲	14.9 ± 10.04	15.70 ± 2.26	16.53 ± 1.00
MF RCSA (%)	–	–	–	–
Right	36.44 ± 5.73■	50.69 ± 21.19■	41.64 ± 7.95■	50.25 ± 7.87
Left	43.26 ± 9.37	52.69 ± 13.87■	41.64 ± 7.95	50.25 ± 7.87
ES RCSA (%)	–	–	–	–
Right	55.75 ± 11.88	76.20 ± 12.28	58.64 ± 8.47	59.99 ± 5.43
Left	54.39 ± 7.75	66.22 ± 15.28	58.64 ± 8.47	59.99 ± 5.43

Compared with Control group on the same side ★P < 0.05; Compared with the diseased side and the healthy side ▲P < 0.05; Compared with ipsilateral ES ■P < 0.05.

**Table 5:** Paravertebral muscle parameters in LDH patients (L5/S1) and control group.

	LDH patient group (L5/S1)		Control group	
	Diseased side	Healthy side	Right	Left
MF-FIR, %	–	–	–	–
Right	25.12 ± 9.31★▲	16.14 ± 5.32	15.48 ± 3.43	15.84 ± 2.82
Left	22.50 ± 8.08★▲	12.89 ± 8.18	15.48 ± 3.43	15.84 ± 2.82
ES FIR (%)	–	–	–	–
Right	26.68 ± 7.28★▲	16.99 ± 5.80	14.67 ± 2.04	14.53 ± 2.41
Left	21.34 ± 5.73★▲	10.6 ± 6.77	14.67 ± 2.04	14.53 ± 2.41
MF RCSA (%)	–	–	–	–
Right	53.69 ± 6.59■	60.28 ± 23.92■	56.35 ± 6.75■	57.24 ± 7.00■
Left	51.64 ± 20.98	62.08 ± 8.71■	56.35 ± 6.75	57.24 ± 7.00
ES RCSA (%)	–	–	–	–
Right	40.63 ± 17.32	44.91 ± 27.94	39.44 ± 8.04	48.48 ± 9.61
Left	44.14 ± 9.74	46.77 ± 22.42	39.44 ± 8.04	48.48 ± 9.61

Compared with Control group on the same side ★P < 0.05; Compared with the diseased side and the healthy side ▲P < 0.05; Compared with ipsilateral ES ■P < 0.05.

### Analysis of the spine–pelvis parameters

The comparison of the spinal and pelvic sagittal position parameters between the experimental and control groups shows that the LL and SS in the LDH (L4/5) group were lower than those in the control group, P < 0.05. The SVA and PT were higher than those in the control group (P < 0.05). The SVA and PT in the LDH (L5/S1) group were higher than those in the control group (P < 0.05), and no statistical significance in other parameters existed between the experimental and control groups (P > 0.05) (Tables 6 and 7).

**Table 6:** Spinal–pelvic sagittal parameters in LDH patients (L4/5) and control group.

	LDH patient group	Control group
SVA, mm	57.51 ± 22.26★	32.07 ± 22.77
TK, °	27.53 ± 9.54	31.54 ± 10.94
LL, °	36.51 ± 11.12★	46.66 ± 10.78
PI, °	46.28 ± 8.23★	41.35 ± 10.09
PT, °	18.13 ± 6.45★	12.09 ± 7.39
SS, °	27.39 ± 8.77★	33.20 ± 7.09

★P < 0.05, the difference is statistically significant.

**Table 7:** Spinal–pelvic sagittal parameters in LDH patients (L5/S1) and control group.

	LDH patient group	Control group
SVA, mm	55.7 ± 29.05★	32.07 ± 22.77
TK, °	30.9 ± 5.87	31.54 ± 10.94
LL, °	45.48 ± 7.95	46.66 ± 10.78
PI, °	45.89 ± 5.93	41.35 ± 10.09
PT, °	17.47 ± 5.76★	12.09 ± 7.39
SS, °	30.36 ± 7.45	33.2 ± 7.09

★P < 0.05, the difference is statistically significant.

### Correlation analysis of the paraspinal parameters with the spinal–pelvic sagittal parameters

In the LDH (L4/5) group, a negative correlation existed between the healthy-side MF FIR and LL (r = −0.461). The healthy-side ES FIR and LL were negatively correlated (r = −0.486). The bilateral MF RCSA was positively correlated with the PT (r = 0.549, r = 0.515). The bilateral ES RCSA was negatively correlated with the SVA (r = −0.579, r = 0.621). A positive correlation existed between the healthy-side ES RCSA and TK (r = 0.614). In the LDH (L5/S1) group, a negative correlation existed between the ES FIR and LL (r = −0.579) (Table 8).

**Table 8:** Correlation analysis between the paravertebral muscle parameters and spinal–pelvic parameters in patients with LDH.

	SVA		TK		LL		PI		PT		SS	
	L4–5	L5–S1	L4–5	L5–S1	L4–5	L5–S1	L4–5	L5–S1	L4–5	L5–S1	L4–5	L5–S1
MF-FIR, %	–	–	–	–	–	–	–	–	–	–	–	–
Diseased side	0.371	− 0.432	− 0.196	− 0.426	0.082	− 0.038	− 0.017	− 0.212	− 0.073	0.118	0.080	− 0.041
Healthy side	0.017	0.029	− 0.089	− 0.412	− 0.461★	− 0.335	− 0.289	− 0.338	− 0.331	0.397	− 0.222	− 0.497
ES-FIR, %	–	–	–	–	–	–	–	–	–	–	–	–
Diseased side	− 0.104	− 0.162	0.011	− 0.179	− 0.275	− 0.153	− 0.219	− 0.065	− 0.335	0.379	− 0.335	− 0.106
Healthy side	0.335	− 0.171	− 0.240	− 0.335	− 0.486★	− 0.579★	− 0.106	− 0.109	0.036	0.374	0.031	− 0.456
MF-RCSA, %	–	–	–	–	–	–	–	–	–	–	–	–
Diseased side	0.036	0.179	0.015	0.006	0.549★	− 0.129	− 0.110	− 0.053	− 0.213	0.338	0.370	− 0.065
Healthy side	− 0.359	0.106	0.343	0.262	0.515★	0.138	0.234	0.021	− 0.126	− 0.018	0.361	0.179
ES-RCSA, %	–	–	–	–	–	–	–	–	–	–	–	–
Diseased side	− 0.579★★	0.103	0.394	0.015	0.435	− 0.144	0.443	0.000	0.189	0.035	0.048	− 0.071
Healthy side	− 0.621★★	0.138	0.614★★	− 0.018	0.266	− 0.021	0.399	− 0.021	− 0.104	0.153	− 0.020	0.115

★P < 0.05, ★★P < 0.01, the difference is statistically significant.

## Discussion

The muscle groups around the spine are collectively called paraspinal muscles, which are mainly divided into the anterior and posterior groups. Quadratus lumborum and psoas major constitute the anterior group, while MF and ES (consisting of iliocostalis, sacrospinalis, and longissimus) constitute the posterior group. The posterior group of paraspinal muscles plays a major role in the overall occurrence and development of LDH. The multifidus muscle in the posterior group, which is the muscle nearest the midline of the lumbosacral paraspinal muscles, includes the internal spinous process, while the longissimus psoas and iliocostalis muscles and the ventral laminae, includes two main functional layers: one is the deep layer to maintain the stability of the lumbar spine, and the other is the superficial layer involved in the rotation of the lumbar spine and the maintenance of LL^13,14^. Yaltırık et al.^3^ found in their experiments that the multifidus and erector spinae innervated at the same level could be significantly affected by the LDH than the psoas major. Therefore, this study was designed mainly for the posterior group of paraspinal muscles. Cooley et al.^15^ found that the degeneration of paraspinal muscles is the main cause of long-term pain and even disability in patients with LDH. Multifidus muscle atrophy is the most sensitive indication of intervertebral disc degeneration. Ranson et al.^16,17^ mentioned that the degeneration of paraspinal muscles is generally manifested as muscle cross-sectional area reduction and fat infiltration. To compensate for the deviation caused by the relative body size of the individual in the muscle CSA, this study referred to the RSCA calculation method of Urrutia et al.^18^ and adjusted the RCSA, that is, RCSA = CSA of the paraspinal muscle/CSA of the upper vertebral body of the responsible segment × 100%. In this study, we found no significant difference in the FIR and RCSA of the bilateral MF and ES in the control group, but the FIR of the paraspinal muscles on the diseased side of the LDH group was higher than that of the healthy side, which is consistent with the results of Kim et al.^17^ Meanwhile, our study showed no significant difference in the FIR of the healthy side of the LDH group and the ipsilateral paraspinal muscles of the control group. This finding also indirectly indicates that the degeneration of the paraspinal muscles in patients with LDH may be related to the compression and inflammatory stimulation of the nerve root on the affected side, suggesting that the degeneration of paraspinal muscles in patients with LDH is not only affected by age, gender, and the degeneration of the paraspinal muscles themselves, but nerve function damage is also one of its degeneration factors. Ng et al.^19^ mentioned in their study that when the spine is rapidly imbalanced, the paraspinal muscles can rapidly maintain the normal force line and dynamic stability of the lumbar spine, among which the multifidus muscles have the fastest response and the largest effect, which are closely related to the innervation of the muscles. Yaltɩrɩk et al.^3^ also confirmed that the degeneration of paraspinal muscles is significantly related to the compression and stimulation of the lumbar intervertebral disc nerves. For the RCSA, another indicator of paraspinal muscle degeneration, we found that the RCSA of the right MF was lower than that of the ipsilateral ES at L4/5 in the control group, while the RCSA of the left MF was similar to that of the ipsilateral ES. The RSCA of the bilateral MF in L5/S1 was higher than that of the ipsilateral ES, and the results indicate that the ES was larger than the MF at L4/5 and smaller than the MF at L5/S1 in the normal population. The combination of this result with human anatomy suggests that the area of MF gradually increases from the upper to the lower level of the lumbar paraspinal muscles in healthy people, while that of ES displays the opposite. Fortin et al.^20^ also found similar results.

Spinal–pelvic sagittal parameters are a quantitative description of the static sagittal alignment of the spine, which allows scholars to understand the compensation phenomenon of the human body to maintain static balance in physiological or pathological conditions from the numerical changes^21^. Scholars at home and abroad have carried out many studies to obtain more standard spinal–pelvic sagittal parameters. However, due to the influence of age, gender, race, region, diet, and other factors, the sagittal spinal–pelvic parameters vary in different countries. According to the previous LL = PI + 9^22^, the sagittal spinal–pelvic parameters of normal people on the basis of PI measurement were as follows: PI = 44.6° ± 9.5°, LL = 48.4° ± −10.8°, SS = 34.4° ± −8.0°, TK = 24.2° ± −9.6°, SVA = 20.5 ± 30.1 mm. In this study, the average SVA value of the control group was larger than the experimental data of other scholars, which may be related to the selection of age. The healthy subjects included in this study were older those in previous studies. Compared with those of the control group, the SVA and PT of the patients with LDH increased. According to Schwab et al.^23^, an SVA of more than 50 mm indicates a sagittal spino–pelvic imbalance, which greatly affects the patient’s condition and quality of life. The average SVA of the patients in our study is greater than 50 mm, which indicates that the LDH patients are in a state of sagittal imbalance. The change in PT value is believed to be one of the compensatory ways for LDH patients to correct this imbalanced state by gradually tilting the pelvis back. This finding is consistent with the results of Ragnics and Endo et al.’s^7,8^ comparative study of patients with degenerative LDH and healthy people. For patients with L4/5 segmental LDH, our study found that LDH had smaller LL and SS, which may also be the result of the patient’s body correcting the imbalance. However, no difference in TK was found between the patient and healthy groups, which is inconsistent with the results of Barrey et al.^6^, and Endo et al.^7^ also found no difference in TK between the LDH cases and normal people. We think that this phenomenon is related to age and the Roussouly classification^24^. For this controversy, the sample size must be expanded, the age and type grouping must be refined, and further study is needed to explore real changes. Previous studies have shown that adults, except for specific sacroiliac joint diseases, generally do not experience changes in PI^22^, which represents the intrinsic morphology. The results of this study show no significant difference in PI values between the control and experimental groups, indicating that LDH does not affect the change in PI.

Panjabi et al.^25^ divided the spinal stabilization system into three subsystems: ① the spine, ② spinal muscles, and ③ the neural control unit. The three systems are independent but closely related to each other. Any change in one system will lead to the destruction of the stability of the spine, while the other two systems have different changes to maintain the stability of the spine. The correlation between the degeneration of paraspinal muscles and spinal–pelvic sagittal parameters has been found in degenerative kyphosis and other diseases^10^. LDH was also investigated in our study, and the results show the following: In the healthy side of LDH (L4/5) patients, the FIR of MF and ES was negatively correlated with LL (r = −0.461, r = −0.486); the RCSA of the bilateral MF and ES was positively correlated with PT (r = 0.549, r = 0.515) and negatively correlated with SVA (r = −0.579, r = 0.621). The ES RCSA was positively correlated with TK on the healthy side (r = 0.614). On the basis of this result, we believe that the MF and the ES are important muscles for maintaining the balance of the spine. When the FIR is high, the proportion of the functional muscles of the paraspinal muscles of the patient is reduced, and the strength is insufficient to maintain the normal physiological curvature of the spine, and the LL decreases, that is, spinal imbalance occurs. In our study, the course of the LDH patients’ disease exceeded 3 months, and the effect of abnormal posture in the acute stage of LDH can be basically excluded. With the prolongation of the disease course, the MF and the ES atrophy to varying degrees, the RCSA of the muscle decreases, and the synergistic effect of the muscles on the spine decreases, followed by the straightness of the lumbar spine and the increase of SVA. To maintain balance, the thoracic spine curvature decreases, that is, the TK decreases, which can compensate for the forward tilt of the spine, but the thoracic spine mobility is small, and the compensation function is very limited. At this time, to maintain the center of gravity of the trunk in the scope of the “economic cone,” the body will start other compensatory mechanisms, including pelvic supination, hip extension, knee flexion, and ankle extension^26^. However, pelvic supination requires the cooperation of the lumbar and back muscles, the RCSA decreases, the PT decreases, the pelvic supination is weak, and balance cannot be maintained with only the compensation mechanism of hip extension, knee flexion, ankle extension, and so on. Of all the patients admitted to the hospital, at least half must use external force to pace. PT is an important parameter for measuring the compensatory mechanism of the pelvis in patients with LDH. The correlation between the PT and RCSA of paraspinal muscles indicates that the degeneration of paraspinal muscles is involved in the compensatory mechanism of LDH patients^26^. When the sagittal imbalance in LDH patients is serious, more compensatory mechanisms are needed. The increase of PT in pelvic rotation is an important manifestation of this compensatory mechanism, but it occurs at the cost of increasing the energy consumption of the body, and the clinical symptoms of patients are aggravated low back pain and decreased quality of life scores. Similarly, due to the positive correlation between the PT and RCSA of paraspinal muscle, we speculate that RCSA can be used to evaluate the severity of sagittal imbalance in patients with LDH. This provides a new reference for evaluating the severity of sagittal balance in patients with limited pelvic posterior rotation due to hip joint lesions, and evaluating the degree of compensatory mechanism by PT or completing a standing spinal X-ray is difficult. In the L5/S1 LDH group, the FIR of the ES on the healthy side was negatively correlated with LL (r = −0.579), which can be explained by the above theory. Therefore, LDH patients exhibited a high degree of fatty infiltration of the MF and ES, and the proportion of functional paraspinal muscles decreased. In the experimental group, the FIR of the diseased side muscle group was higher than that of the healthy side, which is generally consistent with the conclusion obtained by Parkkola et al.^27^. The resultant force generated by the muscle fibers in the lower segmental region accounted for 40–49% of the total resultant force of all muscles^10^, indicating that the degeneration of the low-level multifidus and erector spinae muscles in the lumbar spine greatly reduces muscle function and affects the spinal pelvic sagittal parameters.

For LDH patients, we should pay more attention to the control of the development trend of the disease, strengthen the education of LDH patients, and avoid the impact of long-term disease on the body. At present, the mainstream clinical treatment concept is conservative and surgical treatments. The basic conservative treatment includes bed rest, continuous lumbar traction, drug therapy or acupuncture, massage, and other traditional Chinese medicine treatment methods. When the patient’s symptoms are stable and no longer aggravated with time, patients can be encouraged to start exercise therapy, such as “little Yanfei” and other actions, to strengthen the core muscles of the lower back, which can further improve the clinical symptoms of LDH patients, reduce the frequency of disease, and improve the quality of life of patients. Some patients with a poor conservative treatment should be surgically treated in a timely manner. Individualized treatment plans are designed according to the conditions of the patients. LDH has been treated by posterior nucleus pulposus extraction, percutaneous puncture interventional surgery and transforaminal endoscopic technology, interbody fusion, and interbody non-fusion surgery. Attention must be given to lumbar dorsal muscle function exercise throughout the occurrence, development, and treatment of LDH. In this study, the authors limited the study population to patients with single-level unilateral low LDH and did not pay attention to the effect of upper disc (or multi-level) LDH on paraspinal muscle degeneration (or spinal pelvic sagittal parameters) and the relationship between the two, which must be clarified in subsequent studies.

## Data Availability

All data generated or analyzed during this study are included in this published article.
